# Molecular prevalence and factors associated with *Ehrlichia canis* infection in dogs from the North Pantanal wetland, Brazil

**DOI:** 10.14202/vetworld.2023.1209-1213

**Published:** 2023-06-05

**Authors:** Mariana Elisa Pereira, Darlan Henrique Canei, Matheus Roberto Carvalho, Álvaro Felipe de Lima Ruy Dias, Arleana do Bom Parto Ferreira de Almeida, Luciano Nakazato, Valéria Régia Franco Sousa

**Affiliations:** 1 Postgraduate Program in Veterinary Science, Faculty of Veterinary Medicine, Federal University of Mato Grosso, Avenue Fernando Correa da Costa, Boa Esperança, Cuiabá, Mato Grosso, Brazil; 2 Faculty of Veterinary Medicine, Federal University of Mato Grosso, Avenue Fernando Correa da Costa, Boa Esperança, Cuiabá, Mato Grosso, Brazil

**Keywords:** bacterium, DNA, dog, ehrlichiosis, Pantanal

## Abstract

**Background and Aim:**

Canine monocytic ehrlichiosis is a vector-borne disease caused by the obligatory intracellular bacterium *Ehrlichia canis*, which is distributed across tropical and subtropical regions worldwide. Its prevalence within dog populations is high in municipalities located across the Pantanal biome, but it remains unknown in Barão de Melgaço, Mato Grosso, Brazil. This study aimed to determine the molecular prevalence and factors associated with *E. canis* infection in dogs domiciled in Barão de Melgaço.

**Materials and Methods:**

A cross-sectional study was carried out to investigate the prevalence of *E. canis* infection in 369 dogs from urban and rural areas in Barão de Melgaço, North Pantanal wetland, Brazil. Initially, the dogs were examined, and, through a questionnaire, the risk factors were investigated. Blood samples were subjected to DNA extraction and PCR was performed to estimate the prevalence of *E. canis* infection.

**Results:**

The molecular prevalence of *E. canis* infection in dogs was 42.5% and none of the studied variables were significantly associated with polymerase chain reaction (PCR) positivity (p > 0.05).

**Conclusion:**

The high molecular prevalence demonstrates an increased transmission of the agent across the city. This also indicates that attention needs to be paid to *E. canis* infection and control measures should be introduced to prevent its transmission. The demographic and clinical risk factors commonly associated with *E. canis* infection in this study were not associated with PCR positivity.

## Introduction

Canine monocytic ehrlichiosis is a tick-borne disease caused by the obligatory intracellular bacterium *Ehrlichia canis* [1]. *Ehrlichia canis* is vectored by the brown dog tick *Rhipicephalus sanguineus* sensu lato (s.l.), which infects mature and immature hematopoietic cells of the mononuclear system [2]. In dogs, the disease can present in three phase: acute, subclinical, and chronic. It can lead to clinical signs of depression, lethargy, anorexia, hyperthermia, emesis, cough, dyspnea, mucous pallor, and uveitis [3]. The diagnosis relies on detecting antibodies and is usually based on an indirect immunofluorescence reaction; a polymerase chain reaction (PCR) analysis of blood or bone marrow samples [4], which identifies the DNA of the agent [2]; or by observing morulae in blood smears during the acute phase of the disease [5]. However, the agent is detectable in only 4% of cases [6].

The disease is widely distributed across the world and is frequently reported in tropical and subtropical regions [1, 7–9]. Furthermore, infection by *E. canis* has been reported across all regions of Brazil [8, 10], including Poconé municipality, which is in the Pantanal biome [11, 12]. The Pantanal biome is the largest tropical wetland area in the world (Bolivia, Paraguay, and Brazil) and one of the most important biodiversity biomes. Although *E. canis* infection in dogs has already been reported in this biome, it has not yet been described in Barão de Melgaço, the municipality encompassing the largest extension (99.2%) of this biome.

This study aimed to estimate the molecular prevalence and the factors associated with *E. canis* infection in dogs from rural and urban areas in the municipality of Barão de Melgaço, North Pantanal wetland, Brazil.

## Material and Methods

### Ethical approval and informed consent

This study was approved by the Ethics Committee for Animal Use of the Federal University of Mato Grosso (authorization: 23108.014950/11-5). All owners provided written and informed consent before inclusion of the dogs in the study.

### Study period and location

**Figure-1:**
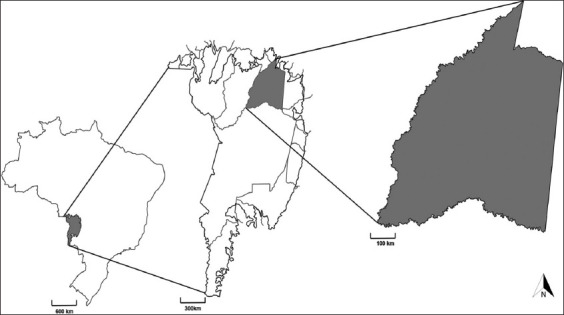
Map of Brazil highlighting the Pantanal biome and the city of Barão de Melgaço, Mato Grosso [Source: https://plataforma.brasil.mapbiomas.org/].

The study was conducted from April 2016 to September 2017. A cross-sectional and descriptive study was conducted in Barão de Melgaço (16°11’40’S and 55°58′03′W), North Pantanal, Brazil (Figure-1)

### Study population and data collection

The minimum sample size was estimated to be 278 dogs. It was based on a dog-to-human ratio of 7:1. The estimated dog population used was 1076 [13], the confidence level was 95%, and the acceptable error was 5%. Samples were collected from 369 dogs in urban and rural areas. Dogs of both sexes, different breeds, and aged 6 months or older were included in the study.

Complete histories, including sex, age, race, function (hunting or companion dogs), environment (rural or urban area), type of housing (indoor or yard dogs), and access to the street, were recorded. A physical examination, which included palpation of the abdomen (splenomegaly) and lymph nodes, oral and ocular mucous membrane color (pale, jaundiced, or normocolored), presence of ophthalmopathy, and presence of ticks, was performed.

Samples were collected from all domestic dogs on a census basis with the agreement of the owners. The dogs were examined for tick infestation and considered symptomatic when they presented with at least one of the following clinical signs: pale or jaundiced mucous membranes, ophthalmopathy, lymphadenomegaly, or splenomegaly. Blood samples were collected by puncturing the external or cephalic jugular vein. The blood was then placed in tubes containing ethylenediaminetetraacetic acid and stored in microtubes at −80°C.

### Detection of *E. canis* DNA by PCR

DNA was extracted from the blood using the phenol/chloroform method [14]. Then, the DNA integrity and the absence of inhibitors were verified by analyzing the DNA samples for endogenous canine globin gene (5’-CAA CTT CAT CCA CGT TCA CC-3’ and 5’-ACA CAA CTG TGT TCA CTA GC-3’), which was then used as an internal amplification control [15].

The DNA detection process used primers E_can0503F (5’-CAG CAA ATT CCA ATC TGC ACT TC-3’) and E_can0503R (5’-GAG CTT CCA ATT GAT GGGTCT G-3’), in which Ecaj_0503 encodes 147 base pairs of a hypothetical protein (E_can0701 system). A PCR analysis was performed, with adaptations, using an Applied Biosystems™ Veriti™ 96-Well thermal cycler (Thermo Fischer Scientific- Model #:9902, Singapore) with 4 μL of dNTP; 2 μL of 10× PCR buffer (Sigma-Aldrich^*^, St. Louis, MO, USA); 0.5 μL of 10 pmol primer; 0.2 μL of Taq DNA polymerase (Sigma-Aldrich); and 17.3 μL of Milli-Q water for each DNA sample. The reaction program consisted of a denaturation cycle at 95°C for 15 min, followed by 40 cycles of denaturation at 95°C for 15 s, annealing at 61°C for 15 s, extension at 72°C for 30 s, and a further extension step at 72°C for 5 min [16].

The amplified products were separated by electrophoresis on 2% agarose gel for 1 h at 60 V, stained with GelRed (Biotium, Hayward, CA, USA), and visualized using a ChemiDoc XRS Molecular Imager (Bio-Rad Laboratories Inc†., Hercules, CA, USA) and Image Lab software (Bio-Rad Laboratories Inc.).

### Statistical analysis

Each independent variable (sex, age < or >1 year, breed, and environment) was related to the dependent variable (PCR positivity) using the Chi-square or Fischer’s exact tests, which were performed using Epi info software version 7.2.4.0 (CDC, Atlanta, GA, USA). The value was considered significant at p ≤ 0.05.

## Results

Of the 369 dogs evaluated in the study, 157 showed DNA amplification for *E. canis*, which was equivalent to a molecular prevalence of 42.5% (range 37.1%–47.2%). A total of 56% were male (207/369), 92.14% were older than 1 year (340/369), 89.9% were of mixed parentage (332/369), 97.3% remained outdoors (359/369), 64.23% lived in an urban area (237/369), and 74.4% had access to the street (271/369). There was no significant association between these variables and the detection of *E. canis* (p > 0.05) by PCR (Table-1).

**Table-1:** Descriptive analysis and its association of sex, age, race, function, environment, and access to the street with *Ehrlichia canis* infection in dogs domiciled in the municipality of Barão de Melgaço, North Pantanal, Brazil.

Variables	Descriptive analysis		Bivariate analysis	
	
	n	PCR+ (%)	Confidence level 95%	p-value
Sex				
Male	207	84 (40.58)	0.83	0.44
Female	162	73 (45.06)	(0.55–1.26)	
Age				
<1 year	29	11 (37.93)	0.81	0.74
>1 year	340	146 (42.94)	(0.37–1.77)	
Breed				
Mixed	332	139 (41.87)	0.76	0.54
Pure	37	18 (48.65)	(0.38–1.50)	
Area of residence				
Rural	132	54 (40.90)	0.9	0.71
Urban	237	103 (43.46)	(0.58–1.38)	
Environment				
Indoor	10	4 (40.00)	0.89	1.00
Outdoor	359	153 (42.62)	(0.24–3.23)	
Access to street				
Yes	271	120 (44.28)	1.31	0.31
No	98	37 (37.75)	(0.82–2.10)	

N=Number of dogs analyzed by PCR, PCR=Polymerase chain reaction

Similarly, no significant associations (p > 0.05) were observed between *E. canis* infection and tick infestation (211/369), clinical signs (226/369), mucosal color (33/369): pale (29/369) or icteric mucous (4/369), petechiae (5/369), ophthalmopathy (30/369), lymphadenomegaly (155/369), and splenomegaly (53/369) (Table-2).

**Table-2:** Descriptive analysis and its association of tick infestation, clinical signs, mucous membranes, ophthalmopathy, lymphadenopathy, and splenomegaly with *Ehrlichia canis* infection in dogs domiciled in the municipality of Barão de Melgaço, North Pantanal, Brazil.

Variables	Descriptive analysis		Bivariate analysis	
	
	n	PCR+ (%)	Confidence level 95%	p-value
Tick infestation				
Yes	211	93 (44.07)	1.15	0.56
No	158	64 (40.50)	(0.76–1.75)	
Clinical signs				
Yes	226	93 (41.15)	0.86	0.56
No	143	64 (44.75)	(0.56–1.32)	
Mucous membranes				
Pale or jaundiced	33	13 (39.39)	0.87	0.84
Normocolored	336	144 (42.85)	(0.42–1.80)	
Petechiae				
Yes	5	1 (20.00)	0.33	0.29
No	364	156 (42.85)	(0.03–3.01)	
Ophthalmopathy				
Yes	30	15 (50.00)	1.38	0.50
No	339	142 (41.88)	(0.66–2.93)	
Lymphadenomegaly				
Yes	155	59 (38.06)	0.73	0.17
No	214	98 (45.79)	(0.48–1.11)	
Splenomegaly				
Yes	53	21 (39.62)	0.87	0.75
No	316	136 (43.03)	(0.48–1.57)	

N=Number of dogs analyzed by PCR, PCR=Polymerase chain reaction

## Discussion

The high molecular prevalence of *E. canis* infection (42.5% of the examined dogs) was similar to that observed in Cuiabá [13], the capital city of Mato Grosso, located 110 km from the study area. This similarity was observed despite the use of distinct diagnostic methodologies. However, the prevalence exceeded that observed by Witter *et al*. [17], which showed positivity for *E. canis* in 23.3% of dogs treated at a veterinary hospital in the capital. The recorded prevalence rates ranged from 2.64% [18] to 76.3% [4] and may be related to the distribution of the vector in the study area, the diagnostic methodology used, and the sampling method [17, 19]. A previous study Costa Jr *et al*. [20] found a higher prevalence of *E. canis* in dogs from rural areas than in those from urban areas, probably due to greater exposure to vectors and lower control of mites in rural areas. However, such an association was not observed in this study (p > 0.05), which is consistent with the results of Da Silva *et al*. [13].

No association was observed between *E. canis* infection and sex, which is consistent with previously reported results [13, 17, 21, 22]. However, Costa Jr *et al*. [20] demonstrated a strong association between seropositivity for *E. canis* and male dogs. This variable was analyzed based on the proportion of males and females present in each study.

The occurrence of infection in dogs was not associated with age (p > 0.05), which agrees with the results of Sousa *et al*. [22] and Paulino *et al*. [23]. Dantas-Torres *et al*. [24] observed an association between dogs under 1 year of age and *E. canis* PCR positivity. It is important to highlight that more than 90% of the dogs were up to 1 year of age, which influenced the analysis. There was no significant association between *E. canis* infection and race, as observed in the previous studies [4, 13], although only 10% of the dogs were purebred. According to Harrus *et al*. [19], German shepherd dogs show severe clinical signs of *E. canis*, although they are no longer susceptible to infection.

Mato Grosso is a region with high tick infestation rates, which is usually associated with high rates of *E. canis* infection [25]. However, no significant association was observed between infection and tick parasitism (p > 0.05). It is worth mentioning that it is a chronic disease, so diagnosis may not be associated with vector infestation [17]. The lack of an association between infection and parasitism by *R. sanguineus*, the biological vector of *E. canis* [23, 24], requires careful analysis because this study did not undertake morphological identification of ticks.

There was no significant association between infection and function (companion or hunting dogs), environment (indoor or outdoor), or access to the street (p > 0.05), which corroborated the results of the previous studies [11, 22, 26].

Clinical signs (such as pallor of the mucous membranes, ophthalmopathies, lymphadenopathy, and splenomegaly) were non-specific and showed no significant association with infection, which contrasted with the findings of Sousa *et al*. [22] who associated pale mucous membranes with *E. canis* infection. This result may be explained by the fact that several dogs were in the subclinical phase of the disease or showed clinical signs of infection by other endemic diseases found in the region, such as visceral leishmaniasis [27, 28].

## Conclusion

To the best of our knowledge, this is the first study to investigate the molecular prevalence of *E. canis* infection in Barão de Melgaço, the municipality occupying the largest area in the Pantanal biome. The results showed a high rate of canine infection in this biome. However, the demographic and clinical risk factors commonly associated with *E. canis* infection in this study were not associated with PCR positivity.

## Authors’ Contributions

VRFS and ABPFA: Designed the study, methodology, review, and editing. MEP, DHC, AFLRD, and LN: Sample collection, molecular examination, and participated in the writing of the manuscript. MEP, MRC, and VRFS: Analysis and interpretation of data and participated in the writing of the manuscript. All authors have read, reviewed, and approved the final manuscript.
